# Inflammatory Response and Immune Regulation in Brain-Heart Interaction after Stroke

**DOI:** 10.1155/2022/2406122

**Published:** 2022-11-16

**Authors:** Lihua Zou, Ruquan Han

**Affiliations:** Department of Anesthesiology, Beijing Tiantan Hospital, Capital Medical University, Fengtai District, Beijing 100070, China

## Abstract

Cerebrocardiac syndrome (CCS) is one of the secondary myocardial injuries after stroke. Cerebrocardiac syndrome may result in a poor prognosis with high mortality. Understanding the mechanism of the brain-heart interaction may be crucial for clinical treatment of pathological changes in CCS. Accumulating evidence suggests that the inflammatory response is involved in the brain-heart interaction after stroke. Systemic inflammatory response syndrome (SIRS) evoked by stroke may injure myocardial cells directly, in which the interplay between inflammatory response, oxidative stress, cardiac sympathetic/parasympathetic dysfunction, and splenic immunoregulation may be also the key pathophysiology factor. This review article summarizes the current understanding of inflammatory response and immune regulation in brain-heart interaction after stroke.

## 1. Introduction

Stroke is a devastating disease that leads to poor neurological outcomes and cardiovascular complications, and cardiovascular complications are also the second leading cause of death after stroke [1]. Cerebrocardiac syndrome (CCS) is a secondary myocardial injury resulting from acute brain injury characterized by myocardial ischemia and/or myocardial infarction and/or arrhythmia. 4.3% of subarachnoid hemorrhage (SAH) patients were complicated with malignant arrhythmia, which significantly increases the risk of death, severe disability, and prolonged hospital stay [2, 3]. In the first three months after ischemic stroke, 4.1% of patients die from cardiac causes, and 19% suffer from serious adverse cardiac events [4, 5]. Accumulating clinical and experimental evidence suggests that underlying mechanisms of brain-heart interaction after stroke are mainly related to hypothalamic-pituitary-adrenal axis (HPA axis), catecholamine surge, sympathetic/parasympathetic regulation, and gut microbiome dysbiosis [1, 6–9]. Recent evidence demonstrates that both inflammatory response and immune regulation mediate brain-heart interaction after stroke [10–13]. Systemic inflammation is independently associated with stroke and major coronary events [14]. Systemic inflammation after stroke may drive subsequent myocardial injury, resulting in release of cTnI, and the level of leukocytes is considered as predictor of cardiac injury following stroke [15]. The immune response following stroke plays a regulatory role in mediating brain-heart interaction [6, 12, 13]. Therefore, this review is to summarize the current understanding of the role of inflammatory response and immune regulation in brain-heart interaction after stroke.

## 2. Inflammation Responses and Immune Regulation Post Stroke

Stroke is the second leading cause of mortality and morbidity worldwide. Stoke is classified as ischemic stroke and hemorrhagic stroke, and the latter includes intracerebral hemorrhage (ICH) and subarachnoid hemorrhage (SAH) [16]. In the acute phase of ischemic stroke, brain injury stimulates the proliferation of microglia and astrocytes. Microglia is the resident inflammatory cell of the brain. M1-type microglia secretes proinflammatory cytokines and chemokines (IL-1*β*, IL-6, TNF-*α*, and IFN-*γ*), as well as nitric oxide synthase (iNOS) and proteolytic enzymes (MMP-9 and MMP-3). Both proinflammatory mediators and reactive oxygen species (ROS) damage endothelial cells and rupture blood-brain barrier (BBB) directly or indirectly. MMP-9 degrades the extracellular matrix of the cerebral microvessel basal lamina, which is consisted by collagen IV, laminin, fibronectin, and interendothelial tight junction proteins. Chen et al., Yenari et al., Zhao et al., and Guo et al. furthermore found that M1-type microglia-induced endothelial necroptosis leading to BBB disruption requires tumor necrosis factor-*α* (TNF-*α*) secreted by M1-type microglia and its receptor, TNF receptor 1 (TNFR1). Anti-TNF*α* treatment significantly ameliorates endothelial necroptosis and BBB destruction and improves stroke outcomes [17–20].

The injured astrocytes, neurons, and oligodendrocytes release brain-derived antigens, including glial fibrillary acidic protein (GFAP), S100, and myelin basic protein (MBP) [21]. Injured endothelial cells and astrocytes produce microvesicles (MVs). The disrupted BBB shows higher permeability, allowing brain-derived antigens and MVs to enter the blood and interact with the peripheral immune system [22]. MVs are formed by outward budding and fission of plasma membrane [23]. The diameter of MV is 100-1000 nm with a lipid bilayer. Lipids, proteins, and RNA, which are important intercellular signal transduction and communicators between the brain and the peripheral immune system, are carried in MVs. In peripheral organs, proteins and microRNAs (miRNAs) in the vesicles regulate the acute cytokine response. The brain-derived antigens and cytokines released into the blood through the BBB indirectly promote the migration of peripheral immune cells to the injured brain tissue that further increase the permeability of the blood-brain barrier, forming a vicious circle [22].

In the subacute and convalescent phase of brain injury, anti-inflammatory cytokines (IL-10, TGF-*β*, IL-4, and IL-3) released by M2-type microglia are conducive to the clearance of necrotic foci, whereas an excessive immunosuppressive response may be related to the risk of infection after stroke [19]. During stroke, the M1-type microglia could transform toM1-type microglia or vice versa, responding to inflammatory signals. Hence, it may be of potential translational value to regulate M1/M2 microglial activation to minimize the detrimental effects and/or maximize the protective role [19]. Astrocytes and oligodendrocytes may also have roles in inflammation in the brain, but little is known regarding the pathology of stroke [24].

The poststroke peripheral immune system initiates complex cellular immune regulation, the imbalance of which is not only an outcome of stroke but also one of the pathophysiological processes aggravating heart and brain injury in stroke patients [6]. T cell is the major respondent in the cellular immune. After the onset of ischemic stroke, circulating type 1 T helper cells (Th1) react with brain-derived antigens (e.g., MBP), then aggravate brain injury, and worsen the prognosis of stroke [21]. Regulatory T (Treg) cells, a subpopulation of CD4(+) with both immunosuppressive and anti-inflammatory properties, could inhibit the production of Th1 cells that balance the production between proinflammatory factors (TNF-*α* and IFN-*γ*) and anti-inflammatory factor (IL-10) [25–29]. Besides, Treg cells may protect the integrity of BBB by inhibiting the elevation of matrix metallopeptidase-9 (MMP-9) and C-C motif chemokine ligand 2 (CCL2) in stroke [6, 30].

Compared with ischemic stroke, there is limited research concerning the immune response in hemorrhagic stroke. In 1964, Crompton firstly described the inflammatory response in SAH patients. Crompton found that monocytes aggregate in the artery endothelial cells near ruptured aneurysms. Inflammatory mediators and immune-related proteins increased in the spasmodic artery endothelial cells [31]. Previous studies have confirmed that inflammation is associated with the poor prognosis of neurological function in patients with hemorrhagic stroke [32, 33]. Erythrocytic products, such as methemoglobin and heme, may activate Toll-like receptor 4 (TLR4) on microglia, which triggers an inflammatory cascade and damages neurons and vascular endothelial cells following subarachnoid hemorrhage [34, 35]. As TLR4 activation, microglia release TNF-*α* and further trigger the upregulation of specific cell adhesion molecules (CAMs) in endothelial cells. CAMs attract macrophages and neutrophils adhering to endothelial cells, then migrating to the subarachnoid space. Subarachnoid macrophages phagocytize the exuded red blood cells (RBCs) via HP-HGB complex, which helps the clearance of hematoma. The binding and clearance of extravesicular free Hgb rely on the identification of Hgb only when it is conjugated to haptoglobin (Hp). Due to the fluidity of the cerebrospinal fluid (CSF) and blood-brain barrier, immune cells are trapped in the subarachnoid space [34, 36–38], where macrophages and neutrophils release numerous inflammatory mediators into the CSF, including endothelin and oxidative free radicals. These inflammatory mediators damage endothelial cells and result in vasoconstriction and arterial stenosis [39]. Cytokines produced by macrophages and neutrophils induce the activation of JAK-STAT and NF-*κ*B, activate the inflammatory signal pathway of adhesion, and increase cell membrane permeability promoting endothelial cell apoptosis [40]. Study in subarachnoid hemorrhage has demonstrated that the prevention of leukocyte extravasation into the subarachnoid space prevents chronic vasospasm [36].

There are fewer studies concerning peripheral immune organs (such as the spleen) after hemorrhagic stroke compared with ischemic stroke. An experiment in intracerebral hemorrhage mice reveals that the volume of brain hematoma is related to spleen atrophy; the larger the volume, the more serious the spleen atrophy. The spleen atrophy reaches a nadir at 48 hours after intracerebral hemorrhage and returns to normal 7-10 days later [6]. The activation of the sympathetic nervous system (SNS)/HPA axis may contribute to spleen atrophy by the rapid decrease of blood flow with increased levels of catecholamine and cortisol. Inhibition of the SNS/HPA axis reverses the spleen atrophy. And yet, the parasympathetic nervous system (PNS) cooperates with SNS in the regulation of the spleen function after stroke [6].

## 3. Inflammation Responses in Brain-Heart Interaction

**Figure 1:**
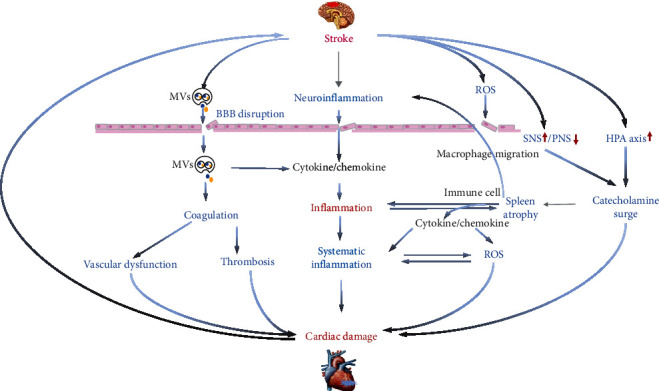
Immune response and inflammation in brain-heart interaction. Local inflammation induces blood-brain barrier (BBB) disruption, oxidative stress, and autonomic hyperactivity. Local inflammation in the injured brain also infiltrates into the systemic circulation. Systemic inflammatory response syndrome (SIRS) injures myocardial cells directly, as well as the interplay between inflammatory response and oxidative stress, cardiac sympathetic/parasympathetic dysfunction, and splenic immunoregulation. Brain-derived microvesicles (MVs) enter into the systemic circulation and then interact with the peripheral immune system. MVs are also involved in thrombus formation. Stroke triggers oxidative stress, and NOX-2 and MCP-1 regulate the release of cytokines/chemokines. Both sympathetic nerve and HPA axis activation cause catecholamine surge. Catecholamines mediate the increase of inflammatory cytokines, as well as spleen atrophy. The spleen regulates the immune response by increasing circulating lymphocytes, cytokines, chemokines, and macrophage migration. SIRS: systemic inflammatory response syndrome; BBB: blood-brain barrier; MVs: microvesicles; SNS: sympathetic nervous system; PNS: parasympathetic nervous system; ROS: reactive oxygen species; NOX-2: nitric oxide-2; MCP-1: monocyte chemoattractant protein-1; HPA: hypothalamic-pituitary adrenal.

Inflammatory response and immune regulation are involved in brain-heart interaction via the direct injury of myocardial cells by inflammatory cells and cytokines, as well as the synergic effect of oxidative stress and autonomic nerve. In addition, the spleen plays a role in brain-heart interaction after stroke. Mechanisms of inflammation in brain-heart interaction after stroke are discussed and summarized in Figure 1.

### 3.1. Direct Cardiac Damage by Inflammation after Stroke

Local inflammation in the injured brain tissue could infiltrate into the systemic circulation reaching peripheral organs such as the heart. The injured endothelial cells and astrocytes in the brain produce MVs, which quickly pass through the highly permeable BBB. Endothelial cell-derived and glial cell-derived proteins (S100B and MBP) and microRNA that carried by MVs regulate the acute response of peripheral cytokines. MVs are categorized as circulation MVs (endothelial MVs, leukocyte MV, and platelet MV) and brain-derived MVs. Circulation MVs stimulate the release of IL-6, inducing coronary artery spasm directly [31] and affecting cardiac diastolic function by indirectly inhibiting NO synthase production [41]. A recent prospective cohort reinforces that high levels of circulating MVs after stroke were associated with worse cardiovascular outcome within 3 years [42]. What is more, the procoagulant activity of platelet MV is 50-100 times higher than platelets. Platelet MV plays a role in cardiovascular thrombosis during inflammatory reaction after brain injury. Surface antigens on platelet MV such as P-selectin and phosphatidylserine (PS) reflect platelet activation and procoagulant by causing platelet homing and thrombus formation [43].

The brain-derived MVs increase rapidly in circulation after brain injury, which also participate in blood hypercoagulability. In addition to acting in thrombosis, brain-derived antigens in MVs activate peripheral immune cells and participate in releasing inflammatory factors and initiating the systemic inflammatory response (SIRS). MicroRNAs in MVs are associate with cardiovascular diseases and identified as potential biomarkers of unstable angina and heart failure [44]. Chen et al. found that decreasing miR-126 affects brain-heart interaction after ischemic stroke by inflammation/oxidative stress [1]. However, stroke releases many microRNAs, and the detailed mechanism of the myocardial inflammatory response mediated by other microRNAs warrants further evidence.

Twenty-one percent of stroke patients develop systemic inflammatory response syndrome during hospitalization [45]. There is an increased production of proinflammatory cytokines after stroke onset, which pass through the BBB into the circulation, inducing systemic inflammation. Brain-derived antigens (such as MBP) activate Toll-like receptors (TLRs) and release cytokines and chemokines [46]. Cytokines initiate the systemic inflammatory response, interacting with chemokines, stress hormones, and the autonomic nervous system [47]. Researches in animal stroke models revealed that lymphocytes secrete transforming growth factor-*β* (TGF-*β*) in response to brain-derived antigens (like MBP). TGF-*β* then triggers the release of IL-6 and decreases MCP-1 and VCAM-1 in vascular adventitia cells. Additionally, TGF-*β* is also involved in myofibroblast transdifferentiation [48–51]. As the systemic inflammatory response initiates, antigen-specific autoimmune response starts, which is characterized by activation of Th1 and TLRs and secretion of proinflammatory mediators. TLR-4 is expressed on the surface of myocardial cells, suggesting inflammation response. Evidence has shown that the activation of TLR-4 and the following release of proinflammatory cytokines (such as TNF-*α* and IL-6) are associated with poor prognosis of heart failure and myocardial infarction [52]. Therefore, cytokines and chemokines may directly act on myocardial cells inducing a myocardial inflammatory response. Myocardium histopathologic studies found that myocarditis occurs in patients with SAH, consisting of an influx of neutrophil granulocytes, lymphocytes, and macrophages, coinciding with myocytolysis and thrombi in intramyocardial arteries [49]. CD68+, CD45+, and myeloperoxidase (MPO) were identified in myocardium of patients who died of SAH [49]. In patients with myocardial infarction, monocyte heterogeneity affects both the extent of myocardial salvage and left ventricular function recovery [53]. Additionally, neutrophils adhere to cardiomyocytes affecting myocyte contraction and inducing cardiac contractile dysfunction via integrin *α*-4 [54, 55]. Macrophages and neutrophils not only cause direct damage to the heart but also induce chronic myocardial injury indirectly via proinflammatory factor secretion and promoting fibroblast differentiation [6]. The infiltrating neutrophils further release cytokines and chemokines, which in turn migrate macrophages from the spleen to myocardial tissue. Recent clinical studies have confirmed that serum macrophage migration inhibitory factor (MIF) may be a potential marker of CCS in severe traumatic brain injury and aneurysmal subarachnoid hemorrhage [3, 56]. In all, both systemic proinflammatory cells and cytokines act on the myocardial cell and induce the local myocardial inflammatory response.

The inflammatory mediators released following stroke may also damage vascular endothelial cells. Damaged endothelial cells trigger fibrin formation and the adhesion and accumulation of platelets. The apoptosis of endothelial cells actives coagulation, due to the increased expression of phosphatidylserine and the loss of surface anticoagulant components Collagen in the subendothelial basement membrane is exposed as the endothelial cell contraction or abscission, which further triggers the adhesion and accumulation of platelets. In addition, TNF-*α* and IL-1 induce the synthesis of tissue factors, and apoptotic endothelial cells also release tissue factors into the bloodstream, further boosting coagulation [40, 57]. However, the mechanism of coronary endothelial cell injury during the systemic inflammatory response following stroke needs further study. In conclusion, inflammation may directly damage myocardial cells and endothelial cells after stroke.

### 3.2. Inflammation and Oxidative Stress

Oxidative stress is a crucial mechanism in BBB disruption and myocardial injury. An experiment in mice confirms that ICH induces significant and progressive cardiac dysfunction by cardiac oxidative stress and inflammation [58]. The expression of myocardial nitric oxide-2 (NOX-2) and monocyte chemoattractant protein-1 (MCP-1) is elevated in the heart tissue following stroke [58]. NOX-2 is a potent source of reactive oxygen species (ROS). Studies have found that NOX-2 participates in cardiac systolic and diastolic function and immune response by the formation of neutrophil extracellular traps (NETs) [5, 11]. MCP-1, i.e., CC chemokine ligand-2 (CCL-2), binds to CC chemokine receptor 2 (CCR-2) and regulates the release of TGF-*β* and infiltration of monocyte and macrophage into the myocardium [13, 59]. The infiltration of inflammatory cells induces myocardial injury by releasing proinflammatory and vasoconstrictor factors [11]. In SAH mice, the expression of MCP-1 and NOX-2 is upregulated, and infiltration of neutrophils and macrophage infiltration around the cardiomyocytes are observed [13]. The decrease of MCP-1 in myocardial cells alleviates macrophage infiltration and improves cardiac function, and antagonizing MCP-1 could also alleviate left ventricular remodeling and heart failure. Therefore, increased expression of MCP-1 and NOX-2 in the myocardium after stroke may suggest a relation to heart failure [10–12]. As above, oxidative stress and inflammatory factors may synergistically participate in myocardial injury after stroke.

### 3.3. Inflammation and Autonomic Nervous System

The heart is directly controlled by the brain through the sympathetic and parasympathetic branches of the autonomic nervous system. Recent evidence shows that the autonomic nervous system interacts with the inflammatory response after ischemic and hemorrhagic stroke. Conversely, autonomic hyperactivity may also result from inflammatory lesions of the autonomic nervous system [60]. Sympathetic nerve activation causes an inflammatory response in the myocardium leading to cardiomyocyte necrosis and cardiac dysfunction [61–63]. The injured neurons and glial cells during stroke release proinflammatory mediators stimulating the posterior hypothalamus and sympathetic nerve to release catecholamines. Catecholamines mediate the increase of peripheral and central inflammatory cytokines. *β*-Adrenoceptors are critical for tissue IL-1*β* induction, while both *α*-adrenoceptor and *β*-adrenoceptors contribute to the installation of plasma cytokines [63]. Catecholaminergic stress and influx of inflammatory cells into the heart cause myocarditis after stroke [49]. Moreover, several studies have found that cholinergic neurons may inhibit acute inflammation, and vagus nerve stimulation attenuates the systemic inflammatory response [62, 64]. The coupling of catecholamine release with parasympathetic dysfunction may regulate the inflammatory response that leads to cardiac insufficiency, thrombosis, and myocardial cell necrosis [5, 61, 65, 66].

### 3.4. Spleen Regulation

The spleen is an important peripheral immune organ, where monocytes, neutrophils, lymphocytes, and NK cells are produced. Lymphocytes from the spleen activate macrophages, and macrophages secrete cytokines and chemokines promoting the migration of inflammatory cells. Shortly after stroke, activation of the sympathetic nervous system leads to an increase in norepinephrine and epinephrine in the systemic circulation. Both norepinephrine and epinephrine have been shown to cause significant splenic atrophy and immune cell outflow from the spleen to peripheral circulation. The spleen regulates the peripheral immune response after ischemic stroke by increasing circulating lymphocytes, proinflammatory cytokines, and chemokines (such as TNF-*α*, MCP-1, IFN-*γ*, IL-6, and IL-2) and may also aggravate the inflammatory response in the acute stage of stroke [67]. In the recovery stage of ischemic stroke, spleen atrophy, T cell proliferation, and inflammatory cytokine secretion are decreased, and immune response is relatively inhibited [1, 68]. In 8 weeks after splenectomy in chronic heart failure patients, left ventricular systolic function was improved, and cardiomyocyte hypertrophy was relieved, indicating that the spleen participates in regulating cardiac function [69]. In ischemic stroke studies, it has been confirmed that splenectomy reduces the migration of inflammatory cells, inhibits the activation of astrocytes, and increases the expression of anti-inflammatory factors [70]. Splenectomy alleviates myocardial inflammatory cell infiltration, decreases the expression of myocardial inflammatory factors, and alleviates the heart failure induced by ischemic stroke [10, 12]. In the mouse model of hemorrhagic stroke, SAH significantly induces ventricular fibrillation and cardiac insufficiency; decreases left ventricular ejection fraction, left ventricular shortening fraction, and heart rate; increases the migration of macrophages and neutrophils to the heart; and elevates the expression of diastolic factors in the heart. Splenectomy of mice before SAH decreases the incidence of cardiac insufficiency and the expression of myocardial NOX-2 and MCP-1, suggesting that SAH may lead to acute cardiac insufficiency, and the secondary immune response may involve in cardiac injury after SAH [11]. Spleen-mediated inflammatory cell infiltration was also involved in the acute and chronic myocardial injury induced by ICH. Experiments in ICH mice also demonstrated that splenectomy alleviates cardiac dysfunction [13]. Therefore, the spleen acts as an important immune regulation role in brain-heart interactions after both ischemic and hemorrhagic strokes.

## 4. Conclusions

Local and systemic inflammatory response after stroke is an important factor in brain-heart interaction. Inflammation could injure myocardial cells directly, and its interactions with oxidative stress, cardiac sympathetic/parasympathetic dysfunction, and splenic immunoregulation are also important factors in the myocardial injury after stroke. This article summarizes the possible immune regulating and inflammation mechanisms after stroke (Figure 1), while further research on the complex mechanism and protective intervention of the heart from inflammatory injury after stroke may emerge.

## Data Availability

Data sharing is not applicable to this review article as no new data was created or analyzed.
